# Using Data Hazards to support safe and ethical digital footprint research

**DOI:** 10.23889/ijpds.v8i3.2279

**Published:** 2023-09-11

**Authors:** Nina Di Cara, Natalie Zelenka, Oliver Davis, Claire Haworth

**Affiliations:** 1 School of Psychological Science, University of Bristol; 2 Jean Golding Institute, University of Bristol; 3 MRC Integrative Epidemiology Unit, University of Bristol; 4 The Alan Turing Institute, London

## Introduction & Background

Health research using digital footprint data often involves the collection and use of large datasets that contain deeply personal information to make inferences about the course and onset of illness. In this context, innovating responsibly is essential for the field to develop safe, trustworthy and, ultimately, ethical research.

The inherent interdisciplinarity of digital footprints research can be a challenge to this aim, with different fields having different ethical norms and standards. As well as this, there has been a strong focus to date on traditional ethical issues such as privacy, which do not necessarily account for the breadth of issues that arise in data science and internet-based work.

## Objectives & Approach

Data Hazards is an open-source project that aims to provide a controlled vocabulary of ethical risks (Data Hazards) that can arise from data science research and its implementation. This vocabulary is presented as a set of 11 Hazard labels (v1.0) each with a visual icon and a set of safety precautions. 

Over three events in 2021-2022 we invited feedback from researchers who volunteered to take part in a Data Hazards workshop (N=15). They varied from PhD students to professors and worked across a range of disciplines, and were asked to discuss the case of mental health prediction from Twitter.

## Relevance to Digital Footprints

Since digital footprint technologies have great potential to pave the way for earlier and more personal medical treatment, it is important for researchers to be able to innovate whilst considering and communicating risk. We can then collaborate to establish effective safety precautions that allow us to maintain research momentum, without compromising safety or trust.

## Results

Based on discussion at the workshops and surveys completed by participants, four main Data Hazards were raised for consideration by the digital footprint research community. These were: 'Lack of Community Involvement' relating to the need to further involve those with lived experience in the development of new technologies; 'Reinforces Existing Bias' due to the potential for automated labelling of ground-truth data to bias training datasets; 'Privacy' given the potential disclosure of sensitive information without consent; and 'Danger of Misuse' due to strong potential for malicious use of such technologies.

Other considerations included the potential psychological risk to those labelling suicide and self-harm content with limited support.

## Conclusions & Implications

The Data Hazards identified provide a means of communicating and clarifying ethical concerns so that they can be more easily addressed in this complex and multidisciplinary field. Further collaboration by the research community to develop and agree appropriate safety precautions would help to build trust in these new technologies before they are deployed in practice. 

